# Effect of Different Levels of Niacin on Serum Biochemical Parameters, Antioxidant Status, Cytokine Levels, Inflammatory Gene Expression and Colonic Microbial Composition in Weaned Piglets

**DOI:** 10.3390/ani12213018

**Published:** 2022-11-03

**Authors:** Zhaobin Wang, Zhenfeng Yi, Qiye Wang, Lanmei Yin, Jun Li, Junyan Xie, Huansheng Yang, Yulong Yin

**Affiliations:** 1 National Center of Technology Innovation for Synthetic Biology, Tianjin Institute of Industrial Biotechnology, Chinese Academy of Sciences, Tianjin 300308, China; 2 Hunan Provincial Key Laboratory of Animal Intestinal Function and Regulation, Hunan International Joint Laboratory of Animal Intestinal Ecology and Health, Laboratory of Animal Nutrition and Human Health, College of Life Sciences, Hunan Normal University, Changsha 410081, China; 3 Key Laboratory of Agro-Ecological Processes in Subtropical Region, Hunan Provincial Engineering Research Center for Healthy Livestock and Poultry Production, Scientific Observing and Experimental Station of Animal Nutrition and Feed Science in South-Central, Ministry of Agriculture, Hunan Provincial Key Laboratory of Animal Nutritional Physiology and Metabolic Process, Institute of Subtropical Agriculture, Chinese Academy of Sciences, Changsha 410125, China; 4 Fujian Aonong Biotechnology Group Co., Ltd., Xiamen 361008, China

**Keywords:** niacin, weaned piglets, antioxidant status, weaning stress, 16S rRNA sequencing

## Abstract

**Simple Summary:**

Niacin, an essential vitamin in weaned pig diets, plays an important role in regulating anti-inflammatory effects and gut health. This study aimed to investigate the effects of niacin supplementation in newly weaned piglets’ diets on inflammation resistance, immunity enhancement, intestinal antioxidant status, and intestinal microbial composition. Studies have found that niacin can significantly improve immunity, antioxidant status and anti-inflammatory effects in newly weaned piglets, better relieve stress damage caused by weaning, improve colonic microbial diversity, and promote intestinal health.

**Abstract:**

Niacin plays an important role in regulating the gut health of weaned piglets. In this study, 48 25-day-old weaned piglets (7.9 ± 0.20 kg) produced by 14 sows (3 to 4 piglets per sow) were randomly divided into 4 groups with 6 replicates in each group and 2 piglets in each replicate. Each group was fed diets supplemented with 22.5 (N1), 30 (N2), 45 (N3), and 75 (N4) mg/kg of niacin, respectively. Samples were taken at 7 and 14 d, respectively. The study shows that changes in niacin levels significantly affected the content of IgG and IgM in the serum (*p* < 0.05). Niacin had a significant effect on antioxidant parameters such as MDA, T-SOD, and CuZn-SOD in the jejunal mucosa of weaned piglets (*p* < 0.05). Moreover, significant differences were observed in the expression of cytokines such as TGF-β, TNF-α, and COX2 in the jejunal mucosa (*p* < 0.05). The 16S rRNA sequencing analysis showed that there were significant differences in the colonic species composition, which were also accompanied by changes in the isovaleric acid content (*p* < 0.05). In conclusion, an appropriate increase in niacin dose based on NRC (2012) has an important role in improving the antioxidant status of weaned piglets, alleviating intestinal inflammation in piglets, improving immunity, and regulating the structure of the microbiota.

## 1. Introduction

Since July 2020, China has banned antibiotics in feed. Pharmaceutical feed additives are no longer allowed. In fact, since 2000, Denmark and other EU member states have voluntarily cancelled all antibiotics in feed. The consequence of this is that within one to two weeks after a piglet is weaned, the growth performance declines rapidly, the growth rates slow down, and the incidences of diarrhea increase. In such a severe situation, finding suitable antibiotic alternatives and evaluating the interaction mechanism between the host, microbiota, and pathogens is of urgent importance.

Niacin, a water-soluble vitamin, is essential in pig diets. The daily nutritional requirement of niacin for weaned piglets weighing 7 to 11 kg is approximately 30 mg [1]. Niacin can be converted into nicotinamide in animals and then synthesized into Coenzyme I (NAD). NAD becomes Coenzyme II (NADP) after phosphorylation by ATP [2]. Both niacin and niacinamide act as vitamins to support the biosynthesis of NAD(P). NAD(P) is a specific coenzyme of many dehydrogenases in animals. It participates in carbohydrate, protein, and fat metabolism and is vital in energy metabolism [3,4,5]. Niacin has a variety of pharmacological effects when administered in super-physiological doses. First, when used to treat the corresponding deficiency, niacin causes a flushing reaction [6]. However, this reaction is not harmful to animals. Later, researchers discovered that niacin can regulate the levels of cholesterol, free fatty acids, lipoproteins(A), and triglycerides in animal plasma, and then, they developed it into a broad-spectrum lipid-modulating drug [7,8,9]. In addition, niacin has a good anti-inflammatory performance [10,11]. Recent studies have found that the pharmacological effects of niacin are mediated by the niacin receptor GPR109A/HCA2 [5,12,13]. Since niacin receptors are expressed in adipocytes, immune cells, and keratinocytes, niacin supplements can reduce the occurrence of inflammatory processes in animals through the action of niacin receptors.

Since the intestinal tracts of piglets are not yet well developed, inflammation is easily induced within two weeks after weaning [14]. However, few reports are available on dietary supplementation of niacin to relieve intestinal inflammation in weaned piglets. Therefore, in view of the good biological functions of niacin, this study aims to investigate the effects of different levels of niacin on serum biochemical parameters, antioxidant status, cytokine levels, inflammatory gene expression, and colonic microbial composition in weaned piglets.

## 2. Materials and Methods

All animal experiments were approved by the Ethics and Animal Welfare Committee, and the care and treatment of the animals met the standards set by the Animal Care and Utilization Committee of Hunan Normal University.

### 2.1. Animals

A total of 48 25-day-old weaning piglets (24 males and 24 females; Duroc × Landrace × Yorkshire) produced by 14 sows (3 to 4 piglets per sow) were randomly assigned into 1 of 4 treatments with 6 replicates per treatment and 2 pens (1 piglet per pen) per replicate for 14-day period experiment. Additional niacin content for each treatment was 22.5 (N1, control), 30 (N2), 45 (N3), and 75 (N4) mg/kg, respectively. The niacin was supplemented in the form of nicotinamide (Royal DSM NV, Shanghai, China). The basal diet was formulated to meet the nutritional recommendation for NRC (2012), and it contained 17.15 mg/kg niacin. The diet composition and nutrient composition analysis are presented in Table 1. Each piglet was placed in a separate nursery barn, with all piglets free to eat and drink during this period. The experiment was divided into two stages. On the seventh day, six piglets (three males and three females) were randomly selected from each group for tissue sampling and sacrificed peacefully (P1); the remaining piglets were sampled after euthanasia on day 14 (P2). Blood samples were collected from the anterior vena cava before slaughter. Then, 15 cm of jejunum was taken and gently cleaned with PBS buffer solution. After that, 2 cm was taken and fixed with 4% paraformaldehyde; the rest of the jejunum was used to collect the mucosa with a slide and put it in liquid nitrogen for quick freezing. Colonic stool samples were collected in a 50 mL centrifuge tube and stored at −80 °C. 

### 2.2. Serum Parameter Analysis

A biochemical analyzer (TBA120FR, Toshiba Medical Systems Corporation, Japan) was used to detect many biochemical indexes such as blood urea nitrogen (BUN), glucose (GLU), aspartate aminotransferase (AST), alanine aminotransferase (ALT), total protein (TP), immunoglobulin M (IgM), immunoglobulin A (IgA) and immunoglobulin G (IgG) in serum. The kit for this experiment was provided by Nanjing Jiancheng Biological Engineering Research Institute (Nanjing Jiancheng, Nanjing, China).

### 2.3. Oxidative Stress Index

The jejunal mucosa samples were homogenized with PBS buffer. The malondialdehyde (MDA), superoxide dismutase (SOD), and total antioxidant capacity (T-AOC) in the samples were detected by the kit. The detection method was provided by the manufacturer (Beyotime Biotechnology, Shanghai, China).

### 2.4. Detection of mRNA Expression by Real-Time RT-PCR

The total RNA of the small intestine was extracted with TRIZOL reagent (Invitrogen, Waltham, MA, USA), after which then the genomic DNA in the sample was removed by DNase I (Invitrogen, Waltham, MA, USA), and cDNA was synthesized by the Prime Script RT Reagent Kit (TAKARA, Kusatsu, Japan). After cDNA dilution, quantitative reverse transcription polymerase chain reaction (qRT-PCR) was performed, and SYBRTM Green PCR Master Mix (Thermo Fisher Scientific, Waltham, MA, USA) was used for real-time quantitative PCR on QuantStudio 5 Real-Time PCR System (Thermo Fisher Scientific, USA).

According to the conserved sequence of related genes of pigs logged by Genbank, we designed the primer with TsingKe Biological Technology Ltd. (Beijing, China) and Primer 5.0 software and synthesized it with Beijing Jingke Biotechnology Co., Ltd., Beijing, China (Table 2).

### 2.5. Cytokines

Jejunal mucosa samples were made 10% homogenate with PBS buffer, and the concentrations of TNF-α and IL-6 were detected according to the method provided by using ELISA kit (Cusabio, Wuhan, China).

### 2.6. Volatile Fatty Acid Detection

One gram of homogenized colon stool sample was put into a 10 mL centrifuge tube. Then, 5 mL of distilled water was added to it and mixed with the vortex for 30 s. The mixture was centrifuged for 15 min in a 4 °C centrifuge (15,000 rpm). This was followed by mixing the supernatant and 25% metaphosphoric acid solution in a ratio of 9: 1. After centrifuging overnight, it was centrifuged in a 4 °C centrifuge (15,000 rpm) for 10 min. The supernatant was filtered with a membrane having a pore diameter of 0.45 μm and transferred into the injection bottle. Colonic VFAs (acetic acid, propionic acid, isobutyric acid, butyric acid, isovaleric acid, and valeric acid) were determined by gas chromatography (Agilent Technologies 7890B GC System, Agilent, Santa Clara, CA, USA). 

### 2.7. Gut Microbiota Sequencing

The total genomic DNA was extracted from a clean colon stool sample. The V3-V4 region of the 16S rRNA gene was amplified with specific primers. Samples were sequenced on the IonS5TMXL platform provided by Novogene (Beijing, China). Using a single-end sequencing method, a small fragment library was constructed for single-end sequencing. Through Reads shear filtering, Operational Taxonomic Units (OTUs) clustering, analysis of the annotation and the abundance of species, alpha diversity analysis, and beta diversity analysis (Beta Diversity), we chose the *t*-test, MetaStat, and LEfSe statistical analysis methods, such as species composition and community structure of the sample difference significance test.

### 2.8. Statistical Analysis

The data were tested for normal distribution and homogeneity of variance, and a one-way analysis of variance was performed on the data that met the analysis requirements. The differences among the groups were regressed through linear and quadratic terms. *p* < 0.05 meant significant difference among the groups; 0.05 < *p* < 0.1 meant there is a trend of significant difference, and *p* > 0.1 signified no significant difference. 

## 3. Results

### 3.1. Serum Biochemical Index

As Table 3 shows, we found that the serum IgG level of the N1 group was significantly lower than that of other groups at the P1 stage (combined, *p* = 0.033; linear, *p* = 0.036; quadratic, *p* = 0.047). In the P2 stage, the IgG level of the N4 group was lower than that of the N1 and N2 groups (combined, *p* = 0.023; linear, *p* = 0.004; quadratic, *p* = 0.266). Moreover, the IgM level of the N4 group in the P2 stage was significantly higher than that in the other groups (combined, *p* < 0.001; linear, *p* < 0.004; quadratic, *p* = 0.051).

### 3.2. Oxidative Stress Index

The detection results of MDA, T-SOD, CuZn-SOD, and T-AOC in jejunal mucosa are shown in Table 4. In the P1 stage, the T-SOD content in groups N2.P1 and N4.P1 was significantly higher than that of the NI.P1 group (combined, *p* = 0.006; linear, *p* = 0.222; quadratic, *p* = 0.399). The level of MDA was significantly higher in the N3.P1 group than that of the N1.P1 group (combined, *p* = 0.130; linear, *p* = 0.544; quadratic, *p* = 0.034). In the P2 stage, the content of T-SOD in N2.P2, N3.P2, and N4.P2 groups was significantly higher than that in the N1 group (combined, *p* < 0.001; linear, *p* = 0.705; quadratic, *p* < 0.001). The CuZn-SOD level in the N2.P2 group was significantly higher than that in the N1.P2 group (combined, *p* = 0.029; linear, *p* = 0.416; quadratic, *p* = 0.038). Lastly, the level of MDA was significantly lower in the N2 group than in the other groups (combined, *p* = 0.002; linear, *p* = 0.349; quadratic, *p* = 0.013).

### 3.3. Gene Expression

As Table 5 shows, the level of niacin in the diet did not affect the gene expression levels of IFN-γ and IL-1β in the jejunum (*p* > 0.05). At the P1 stage, TGF-β (combined, *p* = 0.001; linear, *p* = 0.030; quadratic, *p* = 0.008) and TNF-α (combined, *p* = 0.001; linear, *p* = 0.837; quadratic, *p* = 0.059) gene expression levels in the N3 group were significantly higher than those in the other groups. Compared with groups N2 and N4, the expression level of the COX2 gene in the N3 group at the P2 stage was significantly higher (combined, *p* = 0.018; linear, *p* = 0.489; quadratic, *p* = 0.056). 

### 3.4. Cytokines of the Small Intestine

As Table 6 shows, the TNF-α level of the N3 group was higher than that of other treatments at the P1 stage (combined, *p* = 0.007; linear, *p* = 0.036; quadratic, *p* = 0.231), which was consistent with the trend of TNF-α gene expression level. No effect of dietary niacin on IL-6 concentration was observed during the whole experiment (*p* > 0.05). 

### 3.5. Microbiota and VFAs of Colon Contents

**Figure 1:**
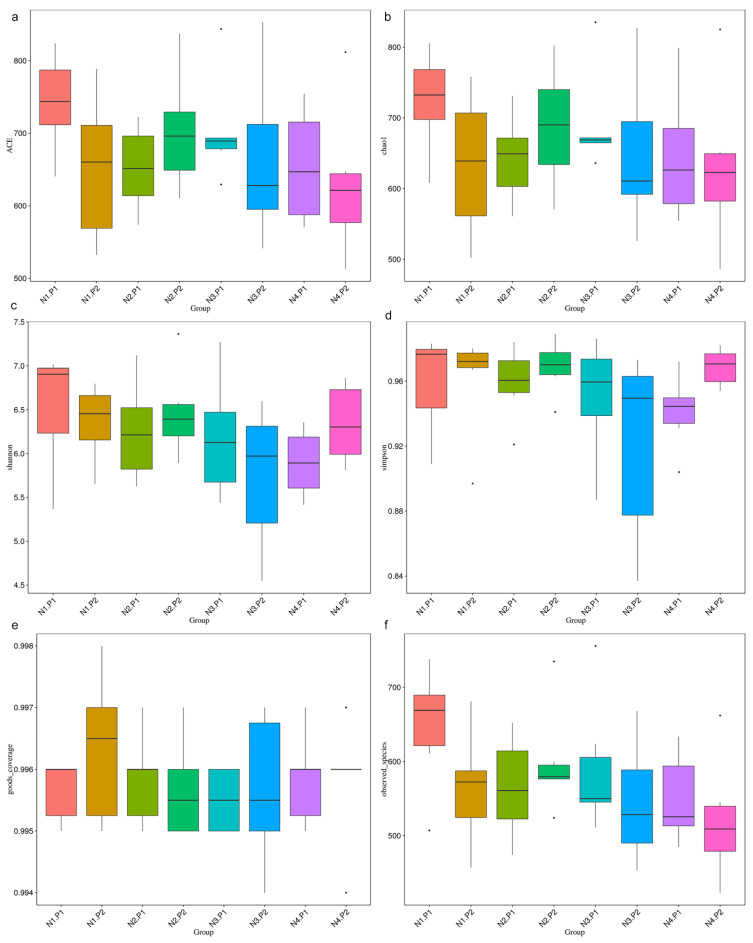
Alpha diversity index analysis. (**a**), ACE index; (**b**), chao1 index; (**c**), Shannon index; (**d**), Simpson; (**e**), goods_coverage index; (**f**), observed_species. P1: the experimental period is 7 days. P2: the experimental period is 14 days. N1: 22.5 mg/kg niacin group. N2: 30 mg/kg niacin group. N3: 45 mg/kg niacin group. N4: 75 mg/kg niacin group. Black dots: abnormal value.

**Figure 2:**
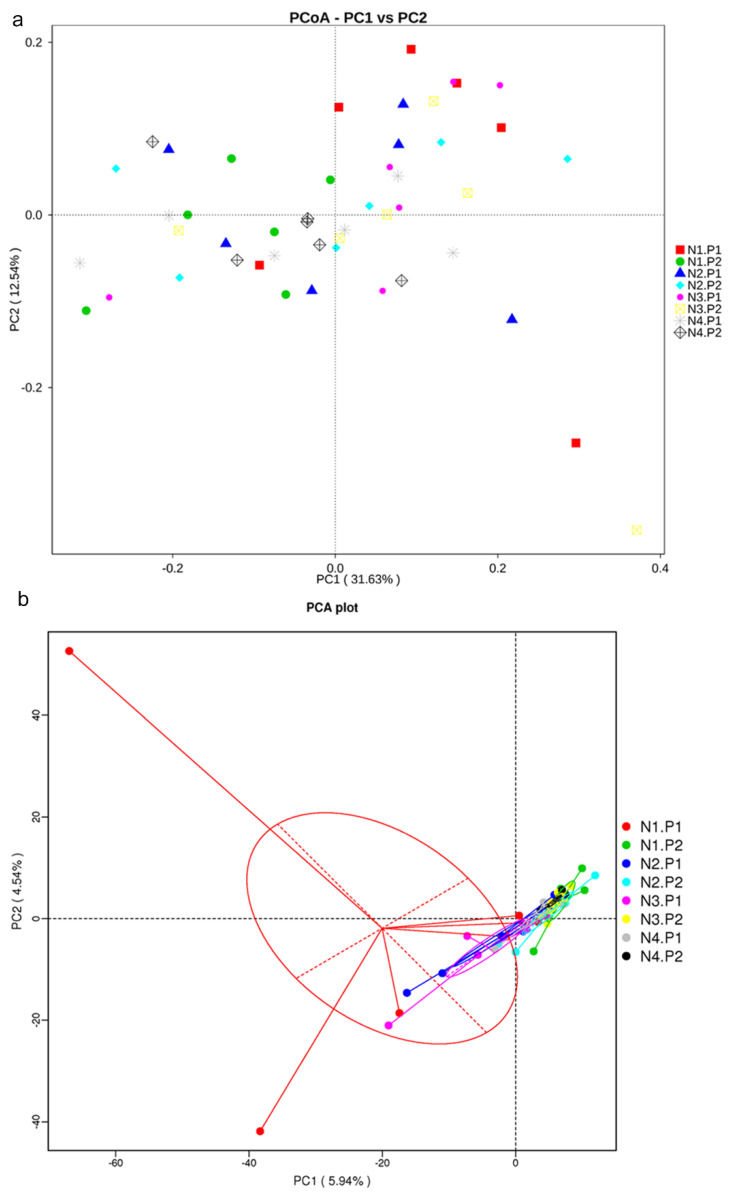
Principal co-ordinates analysis (PCoA) and principal components analysis (PCA). (**a**), PCoA plot; (**b**), PCA plot. P1: The experimental period is 7 days. P2: The experimental period is 14 days. N1: 22.5 mg/kg niacin group. N2: 30 mg/kg niacin group. N3: 45 mg/kg niacin group. N4: 75 mg/kg niacin group.

The sequence of V3-V4 regions of bacterial 16S rRNA genes from colon manure samples, produced 1904380 and 1982345 raw reads in P1 and P2 stages, respectively. OTUs were described at 97% of species similarity, and 15896 and 15085 OTUs were obtained in the samples at the P1 and P2 stages, respectively. The alpha diversity index analysis showed no significant difference (Figure 1a–f; *p* > 0.05). Based on the species annotation results of all samples and the OTUs abundance information, a beta diversity analysis was performed on samples based on weighted Unifrac distance. Figure 2a is drawn with a PCoA. The contribution rates of PCoA1 and PCoA2 to the total variance were 31.63% and 12.54%, respectively. Figure 2b is plotted using PCA. The contribution rates of PC1 and PC2 to the total variance were 5.94% and 4.54%, respectively.

**Figure 3:**
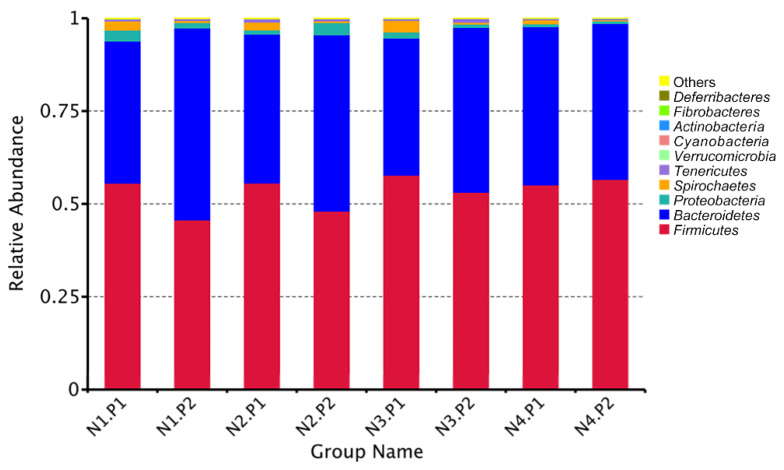
Relative species abundance at phylum level in colonic contents. P1: the experimental period is 7 days. P2: the experimental period is 14 days. N1: 22.5 mg/kg niacin group. N2: 30 mg/kg niacin group. N3: 45 mg/kg niacin group. N4: 75 mg/kg niacin group.

**Figure 4:**
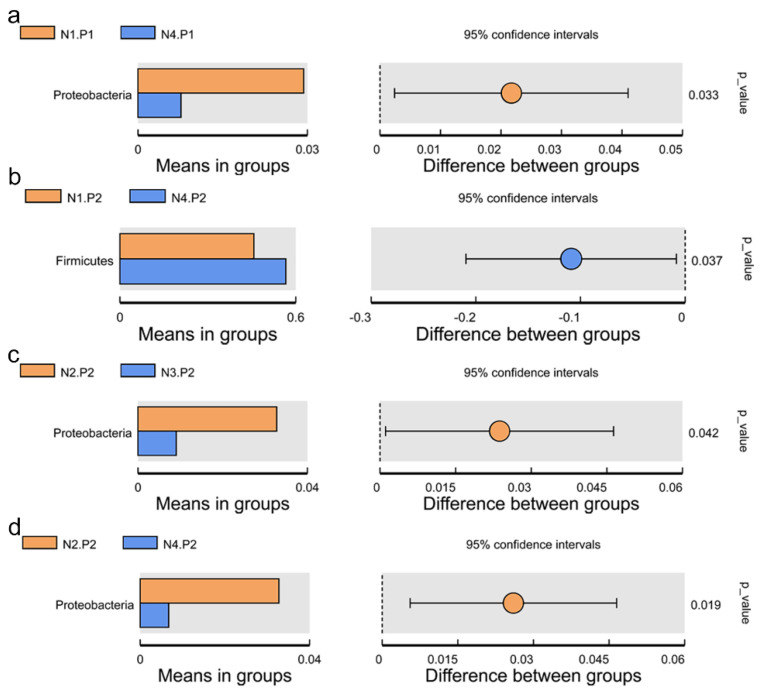
Species between-group difference test (phylum level). (**a**), *t*-test between N1.P1 group and N4.P1 group; (**b**), *t*-test between N1.P2 group and N4.P2 group; (**c**), *t*-test between N2.P2 group and N3.P2 group; (**d**), *t*-test between N2.P2 group and N4.P2 group. P1: the experimental period is 7 days. P2: the experimental period is 14 days. N1: 22.5 mg/kg niacin group. N2: 30 mg/kg niacin group. N3: 45 mg/kg niacin group. N4: 75 mg/kg niacin group.

**Figure 5:**
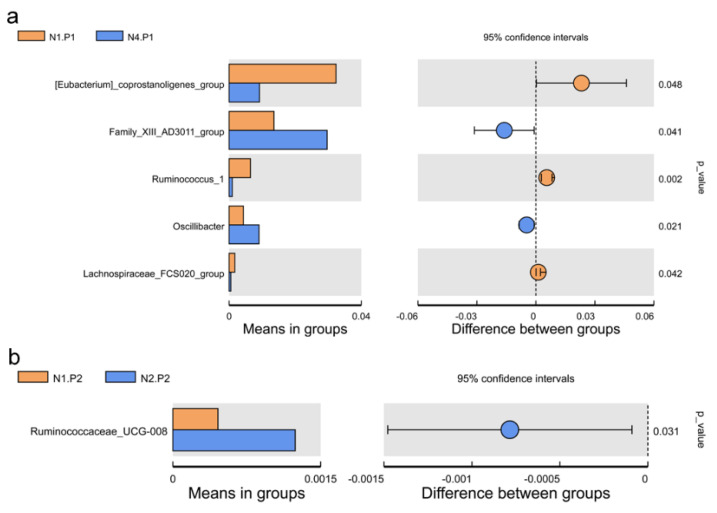
Species between-group difference test (genus level). (**a**), *t*-test between N1.P1 group and N4.P1 group; (**b**), *t*-test between N1.P2 group and N2.P2 group. P1: the experimental period is 7 days. P2: the experimental period is 14 days. N1: 22.5 mg/kg niacin group. N2: 30 mg/kg niacin group. N3: 45 mg/kg niacin group. N4: 75 mg/kg niacin group.

**Figure 6:**
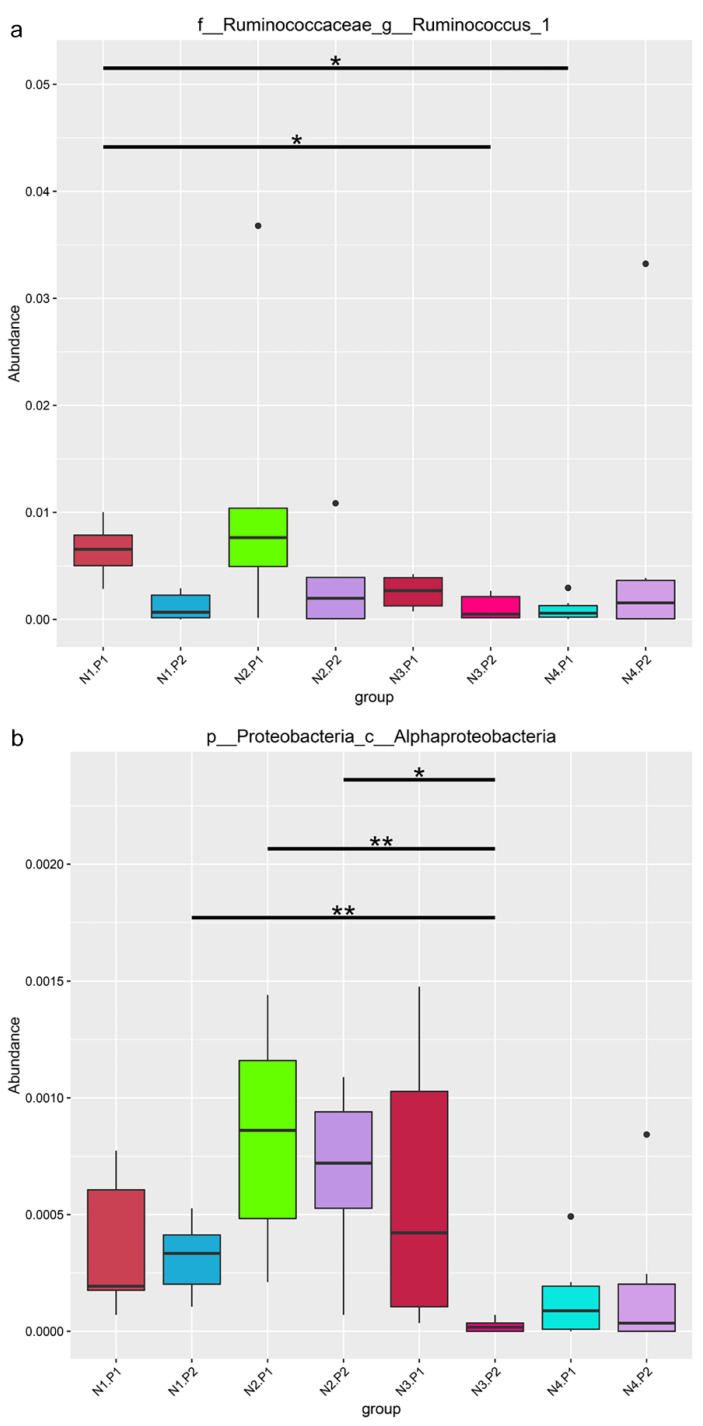
Analysis of significant differences between groups in metastats. (**a**), family_*Ruminococcaceae*_genus_*Ruminococcus*_1; (**b**), phylum_*Proteobacteria*_class_*Alphaproteobacteria*. P1: the experimental period is 7 days. P2: the experimental period is 14 days. N1: 22.5 mg/kg niacin group. N2: 30 mg/kg niacin group. N3: 45 mg/kg niacin group. N4: 75 mg/kg niacin group. Black pots: abnormal value. *: *p* < 0.05; **: *p* < 0.01.

**Figure 7:**
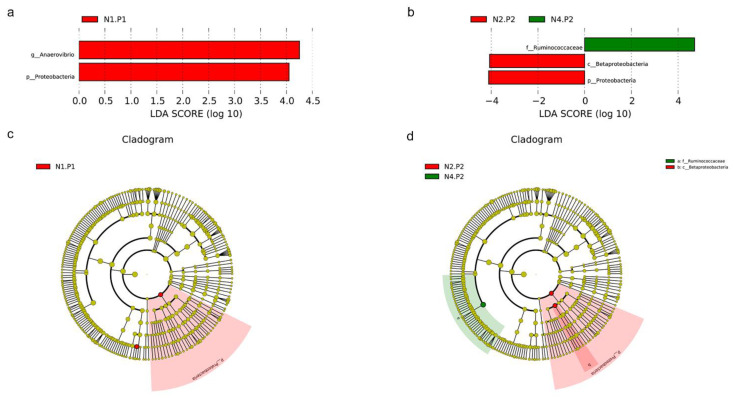
LEfSe analysis. (**a**), histogram of the results of LEfSe among four groups in P1 stage and their respective effect sizes, *p* < 0.05 considered significant; (**b**), histogram of the results of LEfSe among four groups in P2 stage and their respective effect sizes, *p* < 0.05 considered significant; (**c**), cladogram showing taxonomic representation of differences among four groups in P1 stage; (**d**), cladogram showing taxonomic representation of differences among four groups in P2 stage. P1: the experimental period is 7 days. P2: the experimental period is 14 days. N1: 22.5 mg/kg niacin group. N2: 30 mg/kg niacin group. N3: 45 mg/kg niacin group. N4: 75 mg/kg niacin group.

The relative abundance of the sample microorganism at the phylum level is shown in Figure 3. The difference between the groups was analyzed by T-test. At the phylum level, the relative abundance of phylum_*Proteobacteria* in the N4.P1 group was significantly lower than that in the N1.P1 group (Figure 4a; *p* < 0.05). The N4.P2 group had higher relative abundance of phylum_*Firmicutes* than the N1.P2 group (Figure 4b; *p* < 0.05). Furthermore, the relative abundance of phylum_*Proteobacteria* was significantly increased in the N2.P2 group (Figure 4c, d; *p* < 0.05). Additionally, at genus level, the N1.P1 group had significantly higher relative abundance of *[Eubacterium]_coprostanoligenes_group*, *Ruminococcus_1* and *Lachnospiraceae_FCS020_group* than the N4.P1 group (Figure 5a; *p* < 0.05). The relative abundances of *Family_ⅩⅢ_AD3011_group* and *Oscillibacter* in the N4.P1 group were significantly higher than those in the N1.P1 group (Figure 5a; *p* < 0.05). The relative abundance of *Ruminococcace_UCG-008* in the N2.P2 group was significantly higher than that in the N1.P2 group (Figure 5b; *p* < 0.05). The MetaStat method was used to analyze the species abundance between the groups (Figure 6). It was also found that the relative abundance of *Ruminococcus_1* in the N1.P1 group was significantly higher than that in the N4.P1 group at the genus level (Figure 6a; *p* < 0.05). The relative abundance of *Alphaproteobacteria* in the N2.P2 and N3.P2 groups was significantly higher than that in the N4.P2 group at the class level (Figure 6b; *p* < 0.05). The fecal microorganisms in P1 and P2 stages were analyzed by LEfSe (Figure 7), and we found that genus_*Anaerovibrio* and phylum_*Proteobacteria* were biomarkers in the N1.P1 group (*p* < 0.05). Family_*Ruminococcaceae* is a biomarker for the N4.P2 group (*p* < 0.05). Furthermore, the biomarkers of the N2.P2 group are class_*Betaproteobacteria* and phylum_*Proteobacteria* (*p* < 0.05). To better reveal the effect of niacin on the gut microbiota of weaned piglets, we specifically analyzed the changes in the content of volatile fatty acids in the colonic contents of the N1 and N4 groups. As Table 7 shows, we observed that the isovaleric acid content in the N1.P1 group was significantly lower than that in the N4.P1 group (*p* = 0.024).

## 4. Discussion

Niacin is an indispensable essential nutrient in piglet diets. Our previous studies have shown that niacin can influence intestinal morphology and function by affecting cell proliferation of weaned piglets [15]. The latest research shows that it plays an important role in improving the intestinal immunity of weaned piglets [16]. In fish, niacin deficiency reduces the intestinal mucosal immunity and intestinal function of young grass carp [17,18]. Niacin can alleviate inflammation in a GPR109A-dependent manner, thereby effectively alleviating mouse colitis induced by iodoacetamide [19]. Similarly, niacin improves vascular permeability through DP1 mediation, reduces colonic epithelial cell apoptosis, promotes epithelial cell renewal, and inhibits the expression of pro-inflammatory factors in macrophages, alleviating DSS/TNBS-induced mouse colon damage [20]. TNF-α is an inflammatory factor produced mainly by activated monocytes/macrophages. It kills and inhibits tumor cells and is involved in the pathological damage of certain autoimmune diseases [21]. TGF-β is one of the main cytokines that inhibit the function of regulatory T cells and promote the differentiation of pro-inflammatory Th17 and Th9 cells [22,23]. COX2 is an early response gene. The expression of COX2 in healthy animals is low in the gastrointestinal tract, but the expression increases when tissue damage and inflammation are relieved [24]. It plays a key role in the defense and repair of the gastrointestinal mucosa. In this study, the expressions of TGF-β and COX2 in the N3 group were significantly increased, indicating that niacin is exerting a protective effect on piglets under weaning stress. In contrast, TNF-α, an inflammatory cytokine, showed increased expression. One possible reason is that the piglets are in the period of recent weaning, and various external factors lead to the formation of large inflammatory aggregates in the piglets. Short-term niacin supplementation does not yet achieve complete elimination of inflammation.

IgM is the first antibody produced during the immune response. It exists in a secreted form, mainly in the blood [25]. Its unique properties enable it to participate in a variety of pathophysiological processes, including infection, B cell homeostasis, inflammation, and autoimmunity. Natural IgM plays a key role in preventing a series of pathogen infections. It can promote clearance and limit the spread of pathogens, and with the help of the complement component C1q, it can enhance their phagocytosis by phagocytes and increase the presentation of pathogen-derived antigens [25]. Changes in the piglet’s response to infection may lead to chronic inflammation and subsequent autoimmunity. IgM increases the number of B cells and transfers them to the infected area, expressing IgG and thus strengthening the connection with IgM, pathogens, and autoimmunity [26,27]. In this study, IgM and IgG in the N4 group were significantly higher than in the N1 group during the P1 phase. This indicates that piglets supplemented with niacin were able to rapidly improve their autoimmunity in the first week after weaning to cope with the challenges caused by weaning stress. In contrast, at the P2 stage, the immunity of piglets supplemented with high concentrations of niacin was enhanced and their condition gradually recovered, resulting in a significant decrease in the level of IgG produced in the body in response to pathogenic infections.

Many previous studies have pointed out that niacin has a good antioxidant effect, which is reflected in humans, rats, and dairy cows [28,29,30]. In organisms, free radicals act on lipids to cause peroxidation. The end product of oxidation is MDA, which causes cross-linking and polymerization of proteins, nucleic acids, and other cytotoxic life macromolecules. MDA content is an important parameter that reflects the body’s anti-oxidation potential. It can reflect the body’s lipid peroxidation rate and intensity and can also indirectly reflect the degree of tissue peroxidation damage. Superoxide dismutase (SOD) is the main defensive enzyme against free radical damage and scavenges endogenous oxygen free radicals. The characteristics of free radical damage are that it destroys the defense enzyme system, consumes a large amount of SOD, and reduces the activity of SOD. In this study, the P1 phase was the most severe stage of weaning injury in piglets. High dose niacin supplementation could not achieve a rapid increase in antioxidant capacity of piglets. However, at the P2 stage, with increasing doses of niacin, the T-SOD and CuZn-SOD activities in piglets’ jejunum gradually increased, and the antioxidant status of piglets was significantly enhanced, especially with the supplementation of 30 mg/kg. Surprisingly, the MDA level in the N2.P2 group was also high. One possible reason for this is that the jejunum of weaned piglets is still in the repair stage. It may take longer for weaned piglets supplemented with niacin to reach a fully healthy physiological state.

The application of sequencing technology has enriched researchers’ understanding of intestinal microbes. This study shows that *Firmicutes* and *Bacteroidetes* are always the two most important phyla for piglets, which is consistent with previous studies [31,32,33,34]. Previous reports have shown that niacin significantly improves the colonic microbiota and the concentration of volatile fatty acids in hungry weaned piglets. [16]. The vast majority of short-chain fatty acids are produced by indigestible carbohydrates in the intestine through bacterial fermentation, dietary intake, or protein (amino acid) metabolism. Carbohydrates are first hydrolyzed and utilized by bacteria to produce intermediate products, including organic acids such as monosaccharide and oligosaccharide molecules, lactic acid, ethanol, and succinic acid. Bacteria continue to ferment intermediate products to produce short-chain fatty acids as the end product of metabolism. In this study, the level of isovaleric acid in the colon contents of piglets in the N4.P1 group was significantly increased. One possible reason is that high dose niacin supplementation mobilizes the catabolism of leucine in animals, which then increases isovaleric acid production through a series of transamination and enzymatic reactions [35]. In this study, due to the supplementation of niacin, the *Firmicutes* and *Proteobacteria* at the P2 stage increased significantly. *Firmicutes* is one of the largest bacterial groups, most of which are Gram-positive and either spherical or rod-shaped, with cell wall structures. Most of the members of *Firmicutes* are beneficial bacteria, which can promote animal health and play an important role in protecting against pathogens. Several members of *Firmicutes* are believed to be capable of producing short-chain fatty acids, which protect the energy acquisition and immune barrier establishment of newly weaned piglets [36]. The phylum *Proteobacteria* contains a variety of pathogenic bacteria, which are usually associated with intestinal inflammation in weaned piglets [37,38]. As previously reported, the phylum *Bacteroides* consumes oligosaccharides in milk mainly through mucus utilization [39,40]. Due to the change in the weaning diet from liquid milk to solid feed in piglets, coupled with the supplementation of high niacin, the abundance of *Bacteroides* decreased. During the transition period of piglet weaning, due to the change in diet from liquid to solid, the increase in *Firmicutes* and the decrease in the abundance of *Proteobacteria* and Bacteroides may also be an adaptive mechanism for piglets to digest solid feed due to the supplement of niacin. At the genus level, *[Eubacterium]_coprostanoligenes_group* is known as a group capable of converting cholesterol to coprostanol. He et al. found that when piglets were under stress, the level of serum cholesterol and also the abundance of *[Eubacterium]_coprostanoligenes_group* increased significantly [41]. High doses of niacin may alleviate weaning stress through *[Eubacterium]_coprostanoligenes_group* lowering of serum cholesterol levels. Geng et al. found that *Ruminococcus_1* has a gene encoding succinate dehydrogenase, which may be involved in the synthesis of succinate through pathways involved in oxidative phosphorylation and the citric acid cycle [42]. Thus, high doses of niacin may inhibit the production of pro-inflammatory cytokines by reducing the concentration of succinate, thereby reducing the inflammatory response in weaned piglets.

In this study, we explored the effect of niacin in the intestine of piglets based on the variation in its level. The variability between the tested parameters was due to the variability of niacin levels in the diets. This explains the different roles played by niacin in immunity, antioxidant, and anti-inflammatory aspects of piglets and also provides guidance for the application of niacin in weaned piglet diets, especially for anti-stress effects.

## 5. Conclusions

In conclusion, additional supplementation of 45 or even 75 mg/kg niacin in the diet of weaned piglets was effective in improving immunity of piglets and reducing the level of inflammatory factors. Supplementation of 30 mg/kg in weaned piglets can rapidly reshape the antioxidant status in response to intestinal damage caused by weaning stress. Meanwhile, the composition of the colonic microbiota was changed in response to the niacin level.

## Figures and Tables

**Table 1:** Ingredient and chemical composition of piglet diets, as fed basis.

Items	Content
Ingredient, %	
Corn	42.85
Extruded corn	20
Soybean meal	10
Soy protein concentrate	2.88
Whey powder	10
Fish meal	3
Spray-dried porcine plasma	5
L-Lys	0.55
DL-Met	0.12
L-Thr	0.13
L-Try	0.04
Soybean oil	2.55
Limestone	1.08
Dicalcium phosphate	0.7
Choline chloride	0.1
Antioxidants	0.05
Citric acid	0.3
Mineral premix ^1^	0.15
Vitamin premix ^2^	0.5
Total	100
Calculated composition	
CP, %	18.00
ME, kcal·kg^−1^	3,400
Ca, %	0.8
Available P, %	0.36
Niacin, mg/kg	17.15
Lys ^3^, %	1.35
Met ^3^, %	0.39
Met + Cys ^3^, %	0.74
Thr ^3^, %	0.79
Trp ^3^, %	0.22

^1^ Mineral premix per kilogram of feed: 150 mg Fe (FeSO_4_), 100 mg Zn (ZnSO_4_), 30 mg Mn (MnSO_4_), 25 mg Cu (CuSO_4_), 0.5 mg I (KIO_3_), 0.3 mg Co (CoSO_4_), and 0.3 mg Se (Na_2_SeO_3_). ^2^ Vitamin premix supplied per kilogram of feed: 2200 IU vitamin VA, 220 IU vitamin D3, 16 IU of vitamin E, 0.5 mg vitamin K3, 17.5 μg vitamin B12, 3.5 mg riboflavin, 10 mg D-pantothenic acid, 0.05 mg biotin, 0.3 mg folic acid, 1.0 mg thiamine, 7 mg pyridoxine, and 4.0 mg ethoxyquin. ^3^ Standardized ileal digestible.

**Table 2:** Primers used for real-time quantitative PCR analysis.

Genes	Primers	Primers Sequences (5′ to 3′)	Size, bp	NCBI Accession Number
*IFN-γ*	Forward	CCATTCAAAGGAGCATGGAT	146	NM_213948.1
	Reverse	GAGTTCACTGATGGCTTTGC		
*TGF-β*	Forward	CGAGCCCTGGATACCAACTA	164	NM_214015.2
	Reverse	AGGCTCCAGATGTAGGGACA		
*COX2*	Forward	CAAAACCGTATTGCTGCTGA	147	NM_214321.1
	Reverse	AAATTGGGTGATGCCATGTT		
*IL-1β*	Forward	CCTGGACCTTGGTTCTCT	123	NM_214055.1
	Reverse	GGATTCTTCATCGGCTTCT		
*TNF-α*	Forward	ACAGGCCAGCTCCCTCTTAT	102	NM_214022.1
	Reverse	CCTCGCCCTCCTGAATAAAT		
*β-actin*	Forward	AGTTGAAGGTGGTCTCGTGG	215	XM_003124280.5
	Reverse	TGCGGGACATCAAGGAGAAG		

**Table 3:** The serum biochemical parameters in weaned piglets with different niacin concentrations ^1^.

Items ^2^	Dietary Treatment				SEM	*p*-Value	Contrast	
	N1	N2	N3	N4			Linear	Quadratic
P1								
BUN, mmol/L	2.74	3.70	3.58	3.24	0.18	0.261	0.393	0.076
GLU, mmol/L	5.16	5.17	5.66	5.72	0.13	0.292	0.081	0.915
AST, U/L	41.80	42.5	39.20	49.00	1.94	0.333	0.302	0.252
ALT, U/L	20.5	19.00	20.33	21.50	0.85	0.803	0.593	0.464
TP, g/L	34.98	39.93	39.68	38.95	1.14	0.450	0.281	0.233
IgM, mg/dL	13.78 ^b^	11.52 ^bc^	8.83 ^c^	23.43 ^a^	1.37	<0.001	0.001	<0.001
IgA, mg/dL	0.92	0.93	0.77	0.82	0.03	0.239	0.123	0.800
IgG, mg/dL	146.32 ^b^	211.12 ^a^	203.47 ^a^	202.78 ^a^	8.97	0.033	0.036	0.047
P2								
BUN, mmol/L	2.96	2.79	2.61	3.24	0.13	0.347	0.561	0.123
GLU, mmol/L	4.85	4.77	4.44	5.10	0.17	0.609	0.782	0.299
AST, U/L	39.83	43.80	45.33	39.00	1.67	0.488	0.949	0.144
ALT, U/L	17.50	20.33	18.50	18.33	0.90	0.750	0.937	0.433
TP, g/L	32.53	30.35	31.98	31.97	1.25	0.943	0.996	0.687
IgM, mg/dL	11.32 ^b^	11.62 ^b^	14.82 ^b^	21.10 ^a^	1.06	<0.001	<0.001	0.051
IgA, mg/dL	0.80	0.93	0.92	0.87	0.02	0.146	0.344	0.043
IgG, mg/dL	157.80 ^a^	157.55 ^a^	137.24 ^ab^	113.06 ^b^	6.22	0.023	0.004	0.266

^1^ Values are expressed as mean ± SEM, *n* = 6. ^2^ BUN: blood urea nitrogen, GLU: glucose, AST: aspartate aminotransferase, ALT: alanine aminotransferase, TP: total protein, IgM: immunoglobulin M, IgG: immunoglobulin G, IgA: immunoglobulin A. P1: the experimental period is 7 days. P2: the experimental period is 14 days. N1: 22.5 mg/kg niacin group. N2: 30 mg/kg niacin group. N3: 45 mg/kg niacin group. N4: 75 mg/kg niacin group. ^a, b, c^ Values carrying different superscripts are significant statistical differences (*p* < 0.05).

**Table 4:** The oxidative stress index in jejunal mucosa of weaned piglets with different niacin concentrations ^1^.

Items ^2,3^	Dietary Treatment				SEM	*p*-Value	Contrast	
	N1	N2	N3	N4			Linear	Quadratic
P1								
T-SOD	74.54 ^a^	61.94 ^b^	80.26 ^a^	60.62 ^b^	2.61	0.006	0.222	0.399
CuZn-SOD	53.61	46.06	48.17	51.22	2.12	0.622	0.794	0.239
T-AOC	0.99	1.31	1.12	1.71	0.14	0.319	0.130	0.638
MDA	0.32 ^ab^	0.37 ^ab^	0.41 ^a^	0.26 ^b^	0.02	0.130	0.544	0.034
P2								
T-SOD	34.12 ^c^	53.00 ^a^	42.43 ^ab^	38.69 ^b^	1.69	<0.001	0.705	<0.001
CuZn-SOD	27.82 ^b^	36.82 ^a^	28.80 ^b^	27.67 ^b^	1.33	0.029	0.416	0.038
T-AOC	2.41	1.94	2.30	1.49	0.18	0.278	0.154	0.638
MDA	0.34 ^b^	0.63 ^a^	0.32 ^b^	0.39 ^ab^	0.03	0.002	0.349	0.013

^1^ Values are expressed as mean ± SEM, *n* = 6. ^2^ MDA: malondialdehyde, T-AOC: total antioxidant capacity, T-SOD: total superoxide dismutase, CuZn-SOD: CuZn-superoxide dismutase. P1: the experimental period is 7 days. P2: the experimental period is 14 days. N1: 22.5 mg/kg niacin group. N2: 30 mg/kg niacin group. N3: 45 mg/kg niacin group. N4: 75 mg/kg niacin group. ^3^ MDA: nmol/mg of protein, T-AOC: mmol/mg of protein, T-SOD: U/mg of protein, CuZn-SOD: U/mg of protein. ^a, b, c^ Values carrying different superscripts are significant statistical differences (*p* < 0.05).

**Table 5:** The mRNA expression of inflammatory cytokines in jejunal mucosa of weaned piglets with different niacin concentrations ^1^.

Items ^2^	Dietary Treatment				SEM	*p*-Value	Contrast	
	N1	N2	N3	N4			Linear	Quadratic
P1								
*IFN-γ*	1.10	0.62	1.50	1.01	0.16	0.307	0.657	0.982
*TGF-β*	1.00 ^b^	1.26 ^b^	2.66 ^a^	1.42 ^b^	0.18	0.001	0.030	0.008
*COX2*	1.04	1.35	1.53	1.05	0.14	0.613	0.875	0.203
*IL-1β*	1.36	0.67	1.57	0.88	0.21	0.454	0.789	0.994
*TNF-α*	1.08 ^b^	0.61 ^b^	1.92 ^a^	0.70 ^b^	0.15	0.001	0.837	0.059
P2								
*IFN-γ*	1.21	1.61	0.84	1.22	0.17	0.476	0.660	0.971
*TGF-β*	1.05	0.94	0.84	0.81	0.07	0.673	0.234	0.793
*COX2*	1.06 ^ab^	0.91 ^b^	1.54 ^a^	0.66 ^b^	0.11	0.018	0.489	0.056
*IL-1β*	1.06	1.06	0.74	0.78	0.08	0.356	0.121	0.899
*TNF-α*	1.16	1.02	0.98	0.84	0.10	0.788	0.327	0.991

^1^ Values are expressed as mean ± SEM, *n* = 6. ^2^ IFN-γ: interferon-γ, TGF-β: transforming growth factor-β, COX2: cyclooxygenase 2, IL-1β: interleukin-1β, TNF-α: tumor necrosis factor-α. P1: the experimental period is 7 days. P2: the experimental period is 14 days. N1: 22.5 mg/kg niacin group. N2: 30 mg/kg niacin group. N3: 45 mg/kg niacin group. N4: 75 mg/kg niacin group. ^a, b^ Values carrying different superscripts are significant statistical differences (*p* < 0.05).

**Table 6:** The cytokines production in jejunal mucosa of weaned piglets with different niacin concentrations ^1^.

Items ^2^	Dietary Treatment				SEM	*p*-Value	Contrast	
	N1	N2	N3	N4			Linear	Quadratic
P1								
*TNF-α*, ng/mL	49.38 ^b^	47.30 ^b^	63.89 ^a^	54.22 ^b^	2.03	0.007	0.036	0.231
*IL-6*, pg/mL	4.27	3.01	3.72	4.20	0.44	0.762	0.905	0.364
P2								
*TNF-α*, ng/mL	52.17	63.17	56.56	61.64	3.81	0.760	0.551	0.717
*IL-6*, pg/mL	5.85	4.38	3.75	5.63	0.38	0.556	0.839	0.244

^1^ Values are expressed as mean ± SEM, *n* = 6. ^2^ TNF-α: tumor necrosis factor-α, IL-6: interleukin-6. P1: the experimental period is 7 days. P2: the experimental period is 14 days. N1: 22.5 mg/kg niacin group. N2: 30 mg/kg niacin group. N3: 45 mg/kg niacin group. N4: 75 mg/kg niacin group. ^a, b^ Values carrying different superscripts are significant statistical differences (*p* < 0.05).

**Table 7:** The concentration of colon contents in weaned piglets with different niacin concentrations ^1^.

Items	Dietary Treatment		SEM	*p*-Value
	N1	N4		
P1, mg/g				
Acetic acid	3.32	2.69	0.30	0.309
Propanoic acid	1.16	1.18	0.08	0.906
Isobutyric acid	0.11	0.15	0.01	0.125
Butyric acid	0.45	0.50	0.08	0.912
Isovaleric acid	0.22 ^b^	0.27 ^a^	0.01	0.024
Valeric acid	0.17	0.13	0.05	0.179
Total VFA	5.44	4.90	0.44	0.564
P2, mg/g				
Acetic acid	3.46	3.60	0.32	0.833
Propanoic acid	1.22	1.38	0.08	0.347
Isobutyric acid	0.15	0.18	0.01	0.182
Butyric acid	1.06	0.82	0.13	0.379
Isovaleric acid	0.24	0.27	0.02	0.562
Valeric acid	0.22	0.17	0.02	0.289
Total VFA	5.76	6.37	0.44	0.520

^1^ Values are expressed as mean ± SEM, *n* = 6. VFA: volatile fatty acids. P1: the experimental period is 7 days. P2: the experimental period is 14 days. N1: 22.5 mg/kg niacin group. N4: 75 mg/kg niacin group. ^a, b^ Values carrying different superscripts are significant statistical differences (*p* < 0.05).

## Data Availability

All 16S rRNA sequencing data that support the findings of this research have been deposited in the Sequence Read Archive (SRA) and are accessible through the BioProject ID PRJNA879341 (http://www.ncbi.nlm.nih.gov/bioproject/879341, accessed on 12 September 2022).
